# Evaluating the feasibility of using insecticide quantification kits (IQK) for estimating cyanopyrethroid levels for indoor residual spraying in Vanuatu

**DOI:** 10.1186/1475-2875-13-178

**Published:** 2014-05-09

**Authors:** Tanya L Russell, John C Morgan, Hanafy Ismail, Harparkash Kaur, Teunis Eggelte, Folasade Oladepo, James Amon, Janet Hemingway, Harry Iata, Mark JI Paine

**Affiliations:** 1 University of Queensland, School of Population Health, Australian Centre for Tropical and International Health, Pacific Malaria Initiative Support Centre, Herston 4006, Australia; 2 James Cook University, Faculty of Medicine, Health and Molecular Sciences, Queensland Tropical Health Alliance, Cairns 4870, Australia; 3 Liverpool School of Tropical Medicine, Pembroke Place, Liverpool L3 5QA, UK; 4 London School of Hygiene and Tropical Medicine, London, UK; 5 Institute for Tropical Hygiene, Royal Tropical Institute, Amsterdam, The Netherlands; 6 National Vector Borne Disease Control Programme, Ministry of Health, Tafea Province, Vanuatu

**Keywords:** Colorimetric assay, Insecticide residual spray (IRS), Vanuatu, Insecticide quantification kit

## Abstract

**Background:**

The quality of routine indoor residual spraying (IRS) operations is rarely assessed because of the limited choice of methods available for quantifying insecticide content in the field. This study, therefore, evaluated a user-friendly, rapid colorimetric assay for detecting insecticide content after routine IRS operations were conducted.

**Methods:**

This study was conducted in Tafea Province, Vanuatu. Routine IRS was conducted with lambda cyhalothrin. Two methods were used to quantify the IRS activities: 1) pre-spray application of small felt pads and 2) post-spray removal of insecticide with adhesive. The insecticide content was quantified using a colorimetric assay (Insecticide Quantification Kit [IQK]), which involved exposing each sample to the test reagents for 15 mins. The concentration of insecticide was indicated by the depth of red colour.

**Results:**

The IQK proved simple to perform in the field and results could be immediately interpreted by the programme staff. The insecticide content was successfully sampled by attaching felt pads to the house walls prior to spraying. The IRS operation was well conducted, with 83% of houses being sprayed at the target dose (20 – 30 mg AI/m^2^). The average reading across all houses was 24.4 ± 1.5 mg AI/m^2^. The results from the felt pads applied pre-spray were used as a base to compare methods for sampling insecticide from walls post-spray. The adhesive of Sellotape did not collect adequate samples. However, the adhesive of the felt pads provided accurate samples of the insecticide content on walls.

**Conclusion:**

The IQK colorimetric assay proved to be a useful tool that was simple to use under realistic field conditions. The assay provided rapid information on IRS spray dynamics and spray team performance, facilitating timely decision making and reporting for programme managers. The IQK colorimetric assay will have direct applications for routine quality control in malaria control programmes globally and has the potential to improve the efficacy of vector control operations.

## Background

The key vector control strategies used to curb malaria transmission are to provide free long lasting insecticidal nets (LLINs) for the whole population, as well as increasing the coverage of annual indoor residual spraying (IRS) [1]. Essential to the success of these vector control campaigns is implementing strong quality control procedures that monitor programmatic effectiveness in a manner that is simple and sustainable. A major problem for IRS testing is the limited choice of sampling methods for insecticide quantification, and thus this critical factor is not routinely assessed.

The available method for quantifying levels of insecticide sprayed onto surfaces during IRS is high performance liquid chromatography (HPLC) [2]. Cone bioassays are also used to determine the efficacy of the residual insecticide deposited on a wall over time, although they do not quantify the insecticides on the wall [3]. However, both methods are expensive, require highly skilled staff, and have long data turnaround times which significantly impacts on quality control and monitoring processes. For HPLC, a further complication exists for sampling the insecticide from the actual surface. The current World Health Organization (WHO) approved method of sampling involves placing several filter papers (Whatman 5 × 5 cm) at different heights on the walls prior to spraying [4]. While the chemical analysis of insecticides from filter papers has the advantage of being surface independent, they are clearly visible to sprayers, causing bias, and do not allow post spray measurements (important for estimating decay rates). Alternative options for extracting surface residues include taking swabs [5] or by sticky-tape removal [6]. However, while the latter has proven tractable for poorly absorbent wettable powder mixtures such as DDT formulations [6], they both generally suffer from poor extraction efficiency and surface variability.

This study, therefore, focused on evaluating alternative user-friendly and rapid assays for insecticide detection under realistic field conditions in Vanuatu. The range of alternative tests for insecticide quantification under development includes biosensors for DDT and pyrethroid detection [5,7], X-ray and colorimetric tests for cyano-pyrethroids [8,9]. The colorimetric assays, tested here, rely on the chemical detection of cyanide released by alkaline hydrolysis [7,9], and are particularly attractive for field use as they require minimal equipment and operator skills.

In the field site on Tanna Island, Vanuatu, IRS was being conducted by the Ministry of Health with lambda cyhalothrin. These vector control operations formed a component of an ambitious malaria control and progressive elimination programme run by the National Malaria Programmes in Solomon Islands and Vanuatu [10]. As with all spray programmes, were a number of key questions to be answered including: i) has the correct dose of insecticide been applied by the spray teams? ii) have the houses been sprayed uniformly? and iii) how do the insecticide application rates for each area compare? We decided to investigate practical methods for monitoring IRS operations and assist the Ministry of Health to answer these key questions. The Insecticide Quantification Kit (IQK) trialled was a colorimetric assay developed by Kaur and Eggelte [8] for detecting cyano-pyrethroids which are quick and easy to use, and facilitate on-the-spot measurements.

## Methods

### Study site

**Figure 1:**
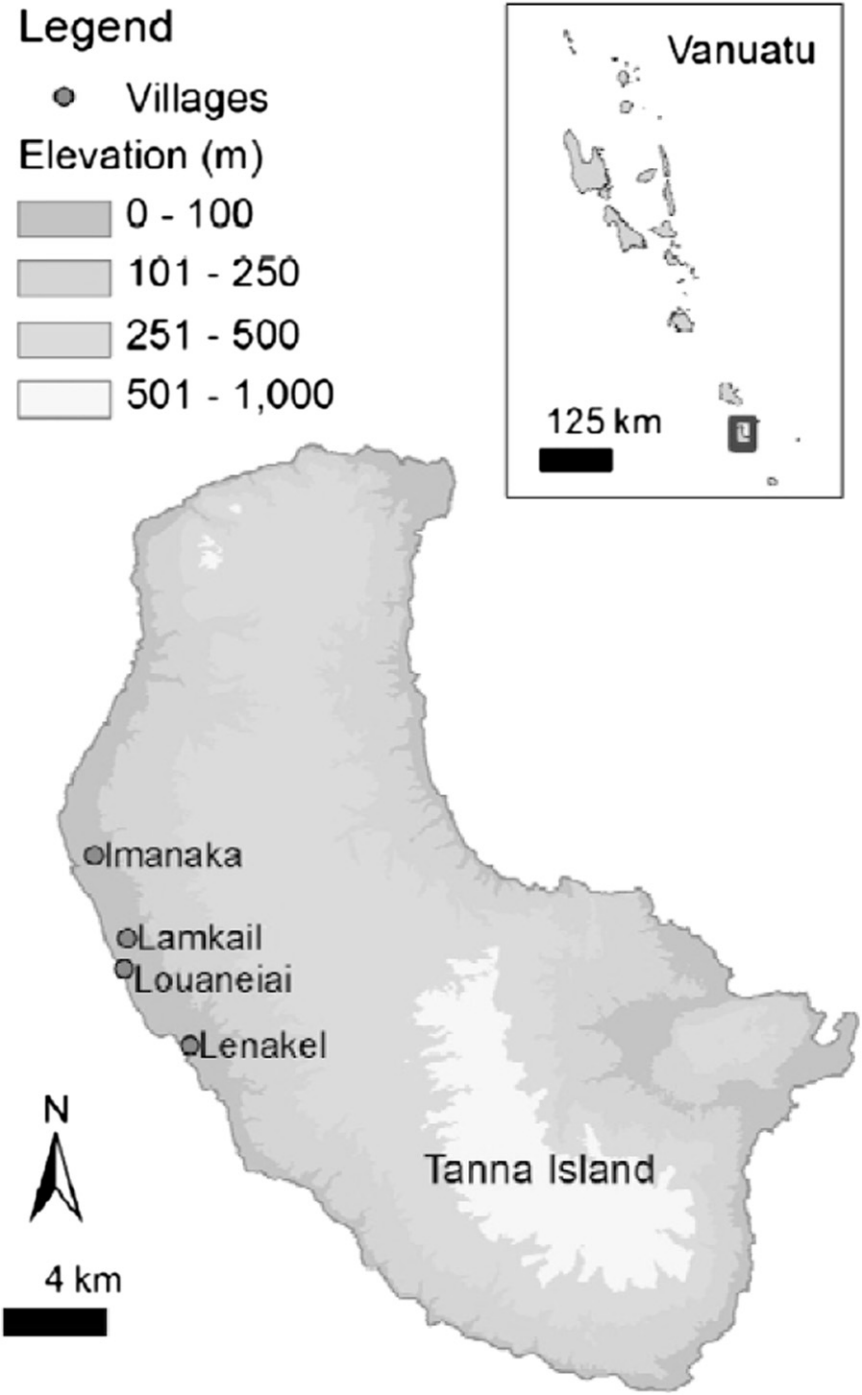
Map of Tanna Island (19.5°S and 169.3°E) in Vanuatu showing the study villages: Imanaka, Lamkail, Louaneiai and Lenakel.

**Figure 2:**
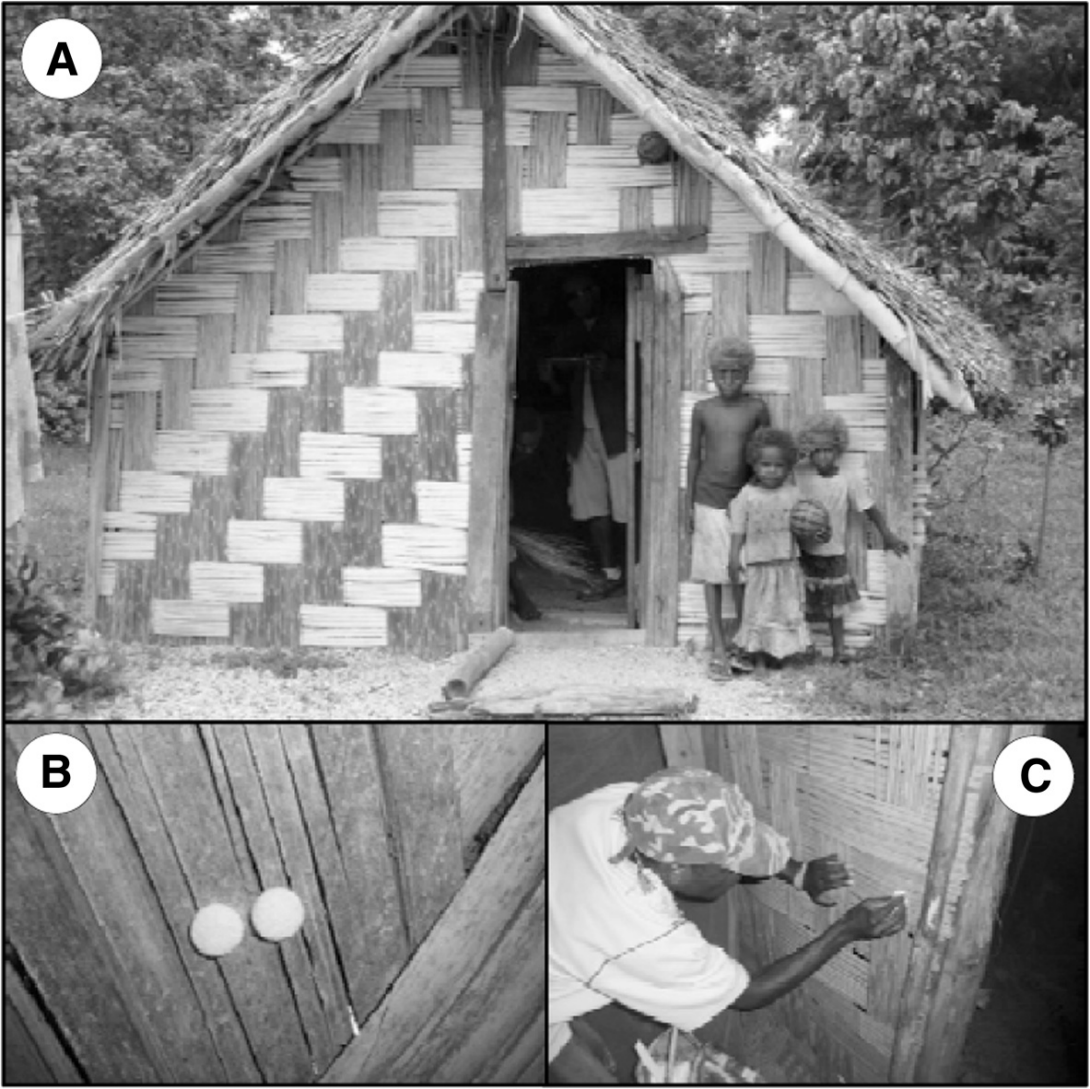
Photographs of study design, including: A) a typical house on Tanna Island; B) pre-spray application of duplicate felt pads; and C) post-spray removal of insecticide with Sellotape.

The study was conducted on Tanna Island (19.5°S and 169.3°E), Tafea Province, Vanuatu in the South West Pacific [11]. Tafea Province has low levels of malaria transmission and is the target for the elimination effort in Vanuatu. Here malaria is transmitted by *Anopheles farauti *[12]. The study villages were Imanaka, Lamkail, Louaneiai and Lenakel (Figure 1). The communities reside in houses built from a range of materials including palm leaf, bamboo, wood or cement (Figure 2A). Routine IRS activities were undertaken by the Vector Borne Disease Control Programme, Ministry of Health, Vanuatu in November 2010.

### Study design

All of the houses in the study villages were sprayed according to WHO guidelines [13]. The formulation used for IRS was ICON 10CS (Syngenta, Switzerland), which is a capsule suspension of lambda cyhalothrin. The target dose was 25 mg active ingredient (AI)/m^2 ^[14]. The amount of insecticide applied to the surfaces was quantified in 30 randomly selected houses across Imanaka (n = 4), Lamkail (n = 11) and Louaneiai (n = 15) villages. Two methods were used to quantify the IRS activities: 1) pre-spray application of small felt pads and 2) post-spray removal of insecticide with adhesive.

### Pre-spray application of felt pads

The first method was a pre-spray application of small felt pads to the walls of each house one day before spraying. This method was similar to the routine WHO method of applying filter paper to the house walls [4]. The pads were 10 mm diameter felt coins, 1 mm thick, obtained from the British Felt Company, Milton Keynes. Being small, the pads were less obvious to sprayers, who were not informed of the test and unaware of the location of the houses chosen or the purpose of the small pads. For each house a total of eighteen pads were stuck on the walls. The pads were attached in pairs (Figure 2B), with three pairs for each of three heights: high (above 2 m), middle (between 1 – 2 m) and low (less than 1 m). From each pair of pads, one was designated for the IQK assay and the other for HLPC analysis. The day after spraying, pads were collected, stuck onto filter paper, labelled and stored in a polythene bag at 4°C before IQK analysis (performed within one month of spraying).

### Post-spray sampling with adhesive

The second method was the post-spray removal of insecticide using the adhesive of Sellotape (Figure 2C) [6] and also the felt pads. For the Sellotape method, a wide (5 cm) transparent strip 20 cm long was pressed firmly onto the treated surface, covering a 100 cm^2^ area. The tape was rubbed well with a cotton ball and then the Sellotape strip was pulled off and stuck to the piece of Whatmann 1 filter paper, labelled and stored in a polythene bag. The felt pads contained a strong adhesive backing and were used as a comparator of insecticide extraction efficiency. The adhesive side of the felt pad was pressed firmly to wall for a couple of seconds, carefully removed and stuck onto filter paper, and stored in a polythene bag at 4°C before IQK analysis (performed within one month of spraying). One sample was taken in each house at random height the day after spraying to measure post-spray concentration.

### Insecticide quantification kits (IQK)

**Figure 3:**
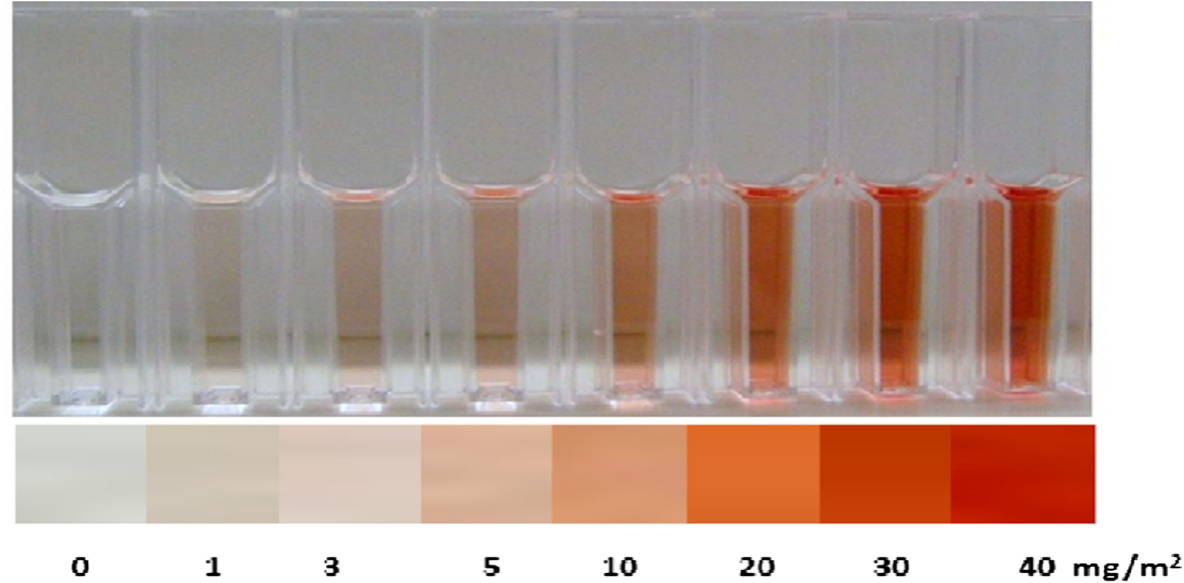
The colour chart used to visually read the results of the Insecticide Quantification Kit (IQK).

The lambda cyhalothrin content was quantified using a colorimetric assay. To conduct the assay, the individual felt pads or a piece of tape were dropped into a labelled glass tube. For tape samples, a 1 cm^2^ piece (approximately the same area as the pad) was cut into small pieces for testing. To the tape or pad samples were added 800 μL reagent A (0.075% potassium hydroxide [KOH] in 90% ethanol), followed by 800 μL reagent B (0.4% 2, 3, 5-triphenyltetrazolium chloride [TTC]; 0.04% 4-nitrobenzaldehyde [PNB] in 90% ethanol). Samples were incubated at room temperature for 15 min with frequent mixing and the reaction stopped by addition of 400 μL reagent C (0.5% acetic acid in 100 ml distilled H_2_O). The concentration of insecticide, indicated by the depth of red colour, was estimated using a colour chart (Figure 3). After the nine individual replicates from each house were read to estimate the overall spray pattern, the samples were pooled into a single tube to provide a combined average, which was taken as an estimate of overall spray quality in each house.

The colour chart was prepared by spiking Whatman No. 1 filter papers cut to 1 cm^2^ with the active ingredient, lambda cyhalothrin. A 0.1 mg/ml stock solution of lambda cyhalothrin was prepared in 100% methanol. The filter papers were spiked with the stock at volumes of 1, 3, 5, 10, 20, 30 and 40 μl, and taken through the IQK procedure to provide the red colour range equivalent to wall spray rates of 1, 2, 3, 5, 10, 20, and 40 mg/m^2^ respectively. A zero control was prepared with 100 μl of methanol. To produce the chart, the dilution range was transferred to clear plastic cuvettes (1 cm light path), and a picture taken against a white background. After importing into Microsoft Powerpoint, representative red areas for each dilution were cropped and cut and pasted to produce a colour strip with depths of red colour representative of each spray rate. The results were classified as: severly underdosed (0 – 3 mg AI/m^2^), mildy underdosed (5–10 mg AI/m^2^), target dose (20 – 30 mg AI/m^2^) and overdosed (≥40 mg AI/m^2^).

### Cut-off point for effective spraying

Duplicate samples were taken for HPLC analysis of insecticide content. However, the pads contained components that interfered with the chromatographic measurements of lambda cyhalothrin. Thus it was not possible to make colorimetric versus HPLC comparisons.

Therefore, to compare the quantified insecticide levels with the potential efficacy of lambda cyhalothrin entomologic bioassays were performed. The WHO cone bioassays [4] were performed on Whatman No. 1 filter papers prepared as for the colour chart with lambda cyhalothrin concentrations equivalent to 0, 1, 3, 5, 10, 30 and 40 mg/m^2^. Four replicates of 25 susceptible *Anopheles gambiae* (Kisumu strain) were exposed for 30 minutes per concentration before being transferred to a clean holding cup. The 1 h knockdown and 24 h mortality was measured. After the bioassay, 1 cm^2^ from each filter was tested by IQK and compared to the colour chart for a visual readout. It is important to reiterate that the LC_80_ value was an arbitrary value used as a qualitative replacement for HPLC quantitation. As such it provided a useful biological reference point for the IQK.

### Statistical analysis

The insecticide content of felt pads applied pre-spray at different heights on the walls—high (>2 m), medium (1 – 2 m) and low (<1 m) —were compared using a generalized linear model (GLM) with a normal distribution. The comparative efficacy of each technique used to sample insecticide was investigated using Pearson’s correlation coefficient. Here, the correlation between the pre-spray felt pads (pooled data) and the post-spray tape and felt pads was compared. For the mosquito survival data, a probit regression was used to determine the concentration of lambda cyhalothrin required for 50%, 80% and 90% mortality (lethal concentration: LC_50_, LC_80_ and LC_90_). All analyses were conducted using R statistical software (ver.2.14.2).

## Results

### House structure

The study houses were randomly selected and were representative of houses within the community. The majority of the houses (n = 23) were constructed from bamboo. The remaining houses were constructed from: bamboo plus iron sheets (n = 1), cement (n = 1), palm leaf (n = 1), plywood (n = 2), timber plus cement (n = 1), and wood (n = 1).

### Pre-spray application of felt pads

**Figure 4:**
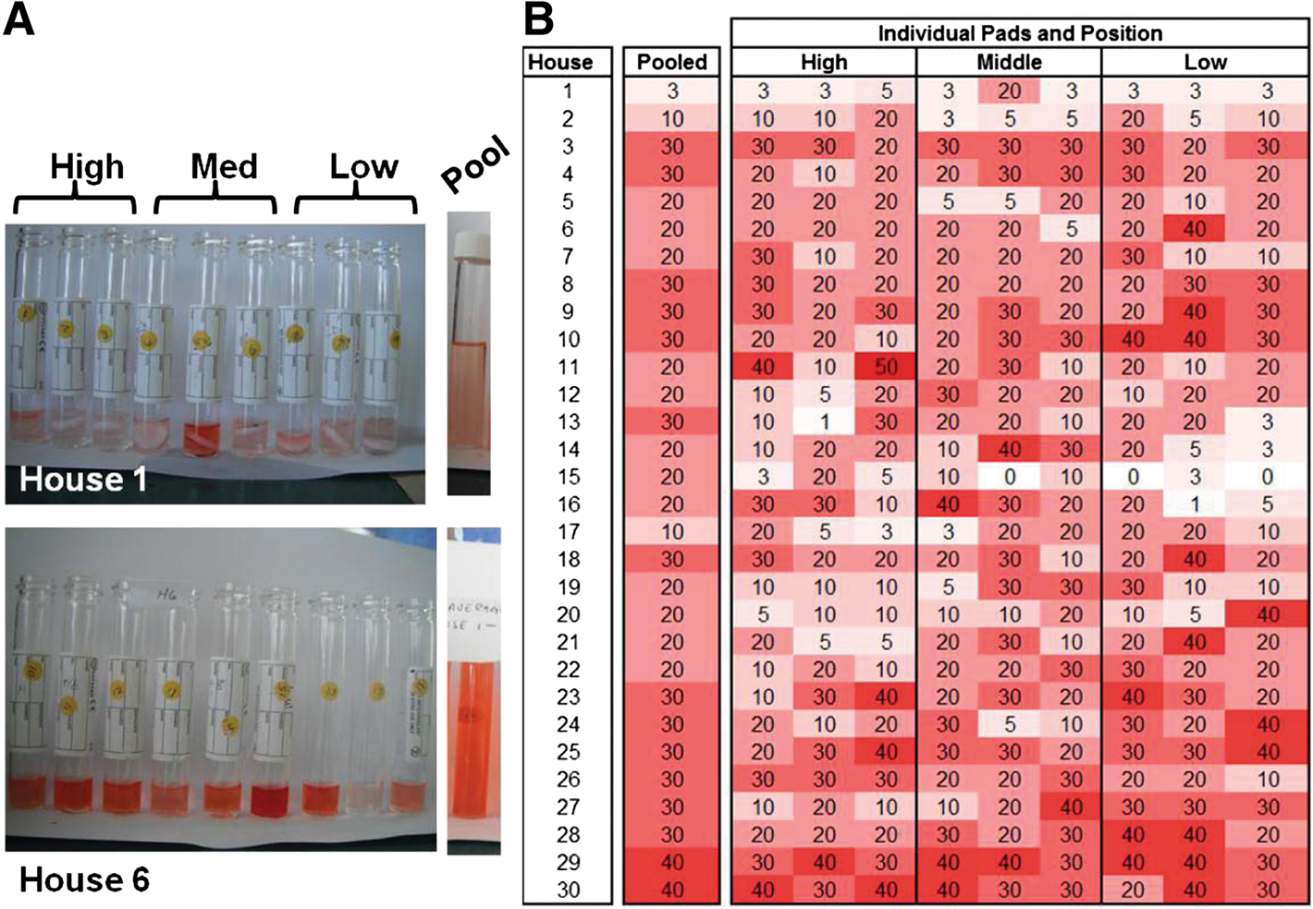
**Results of IQK tests. (A)** Examples of individual colour reactions for pads from a poorly sprayed (House 1) and a well sprayed (House 6) house. All nine reactions were pooled to provide an average spray dose (Pool). **(B)** Table and heat map of Tanna houses sprayed with lambda cyhalothrin. Results for each replicate reaction and the pooled average are displayed for each house. The numbers are the colour chart readings in mg/m^2^; the intensity of red is a visual indication of spray levels.

Attaching felt pads to the house walls prior to spraying proved to be a useful method for sampling the insecticide content. The pads were reasonably small in size and as they had not been used before were unlikely to have caused behavioural bias of the spray team. A total of 270 pads (n = 9 replicates from 30 houses) were screened for insecticide content with the IQK. The reactions proved simple to perform in the field and results could be immediately interpreted by the programme staff. Importantly, houses that were poorly sprayed (House 1) or well sprayed (House 6) were immediately apparent (Figure 4A). Furthermore the dynamics of spraying could also be easily monitored. In House 1 for example, only one replicate at medium height (1 – 2 m) was well sprayed, while House 6 showed consistently good levels of spraying across most of the house.

To support programmatic decision making, a heat map comparing all the replicates from each sampled house was easily prepared in Microsoft Excel (Figure 4B). It is evident from the colour variability in the heat map that heterogeneity in the spraying does occur. Nonetheless, overall the IRS operation was well conducted with the majority, 83.3% (n = 27) of houses being sprayed at the target dose (20– 30 mg AI/m^2^). From the remaining houses, 6.7% (n = 2) were overdosed, 6.7% (n = 2) were mildly under-dosed and 3.3% (n = 1) were severely under-dosed. The samples which were placed at high (>2 m), medium (1 – 2 m) and low (<1 m) positions on the walls were compared and there was no significant difference in the insecticide content (*β* = 1.133, se = 0.807, *p* = 0.162). The average reading for samples that were placed high, medium and low were 18.7 ± 1.1, 20.9 ± 1.1, and 21.7 ± 1.2 mg AI/m^2^, respectively. After the pads had been sampled by IQK, the nine reactions were pooled to provide a qualitative assessment of the household average spray rate (Figure 4A).

### Post-spray sampling with adhesive

**Figure 5:**
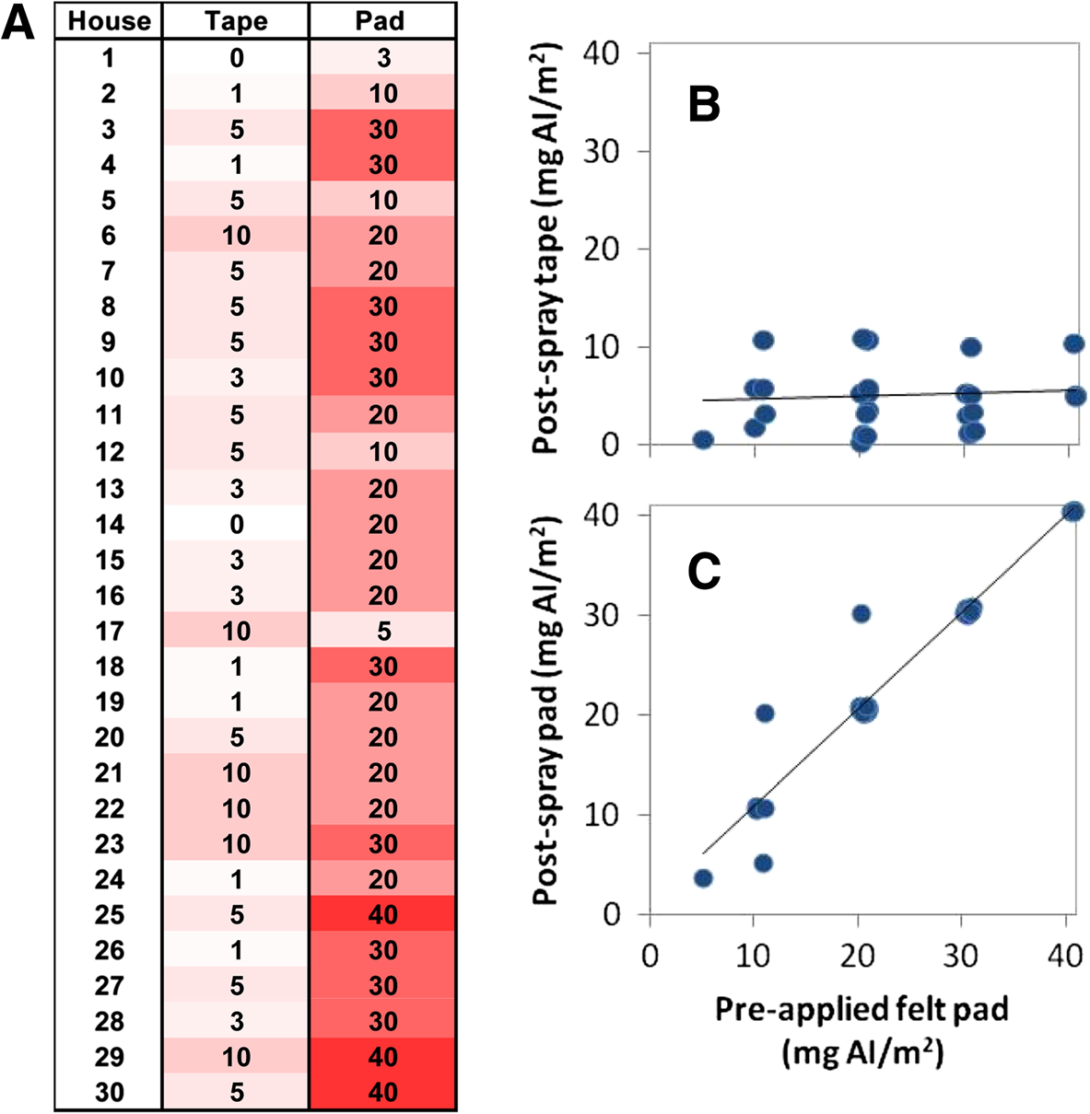
**Results of IQK tests conducted with two methods of post-spray sampling, being the adhesive of Sellotape and felt pads. (A)** Table and heat map of results for each house sampled. The efficacy of post-spray sampling with **(B)** Sellotape and **(C)** felt pads was correlated against the reference method (x-axis): felt pads applied to the surface pre-spray, pooled average for each house. There are less than 30 points as several are overlapping measurements, thus scatter was added to the points when plotting.

The samples collected 1 day post-spray with the Sellotape and the felt pad adhesive were also analysed with IQK (Figure 5A). The results for each method were compared against the pooled felt pads applied pre-spray, for which the average reading across all houses was 24.4 ± 1.5 mg AI/m^2^. The results for the Sellotape samples did not correlate with the results from pre-applied felt pads (*r* = 0.061, df = 27, *p* = 0.752; Figure 5B), with the insecticide content being much lower at 4.7 ± 0.6 mg AI/m^2^. By contrast, the samples collected post-spray with the pad adhesive produced strongly correlating results with pre-applied felt pads (*r* = 0.906, df = 28, *p* <0.0001; Figure 5C) and the average insecticide content was similar at 23.3 ± 1.7 mg AI/m^2^. This is consistent with the fact that the extraction efficiency of Sellotape is low in comparison with pad adhesive (~10% vs ~80% respectively), recommending the pad adhesive for post spray sampling.

### Cut-off point for effective spraying

**Figure 6:**
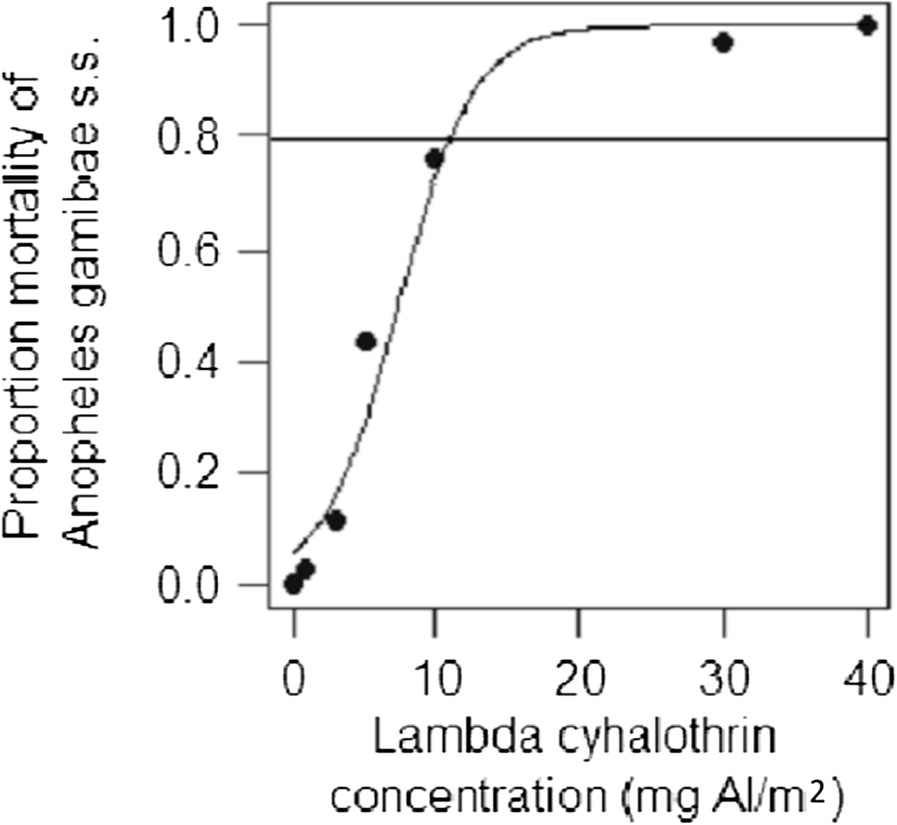
**The 24 h mortality of *An. gambiae s.s. *mosquitoes exposed to different concentrations of lambda cyhalothrin.** Mosquitoes were exposed to the treatments for 1 h in standard WHO cone bioassays. The sigmoidal model was fitted to the data using probit regression.

After *An. gambiae* were exposed to lambda cyhalothrin for 30 min (Figure 6), the LC_80_ was calculated as 10.7 ± 1.0 mg AI/ m^2^. Notwithstanding the limitations of bioassays, which do not quantify insecticide levels, the LC_80_ represents the concentration required to cause 80% mortality and regarded as the cut-off point for effective treatment [15]. These laboratory bioassays confirm that the spray operations were likely to be effective, The LC_50_ and LC_90_ for the bioassays were calculated as 5.9 ± 1.1 and 15.1 ± 1.1 mg AI/m^2^, respectively.

## Discussion

Indoor residual spraying is a highly effective vector control method, involving the coordinated spraying of the interior of houses with insecticides. Importantly, all four classes of WHO approved insecticides can be used for IRS [13], unlike insecticide-treated materials which are limited to pyrethroids [16]. Thus, the use of IRS is likely to increase as resistance to pyrethroids escalates [17]. To ensure successful IRS operations it is essential to implement quality control procedures, but this is rarely done because of the limited choice of methods available for quantifying insecticide content in the field. Here, the practical use of a simple colorimetric field assay, initially developed for ITNs by Kaur and Eggelte [8], has been examined for quantifying insecticide content of IRS. Similar colorimetric assays have been developed for the quantification of pyrethroids [7,9,18] and DDT [5,7], as well as detecting DDT resistance in *Aedes aegypti *[19]. The potential outcomes of the colorimetric assay were matched against measurements of mosquito mortality from laboratory bioassays. The target dose of ICON 10CS recommended by the manufacturer (25 mg AI/m^2^) [14] was well above the LC_80_ for *An. gambiae*, providing room for the insecticide content to decay over time [20].

The biggest obstacle to be overcome when adapting the IQK for monitoring IRS, was to develop a method to accurately sample insecticide from the walls of sprayed houses. It was important to consider the range of house materials which are treated globally and include: plaster, brick, mud, bamboo and straw. The current WHO recommendation is to place filter papers on the walls prior to spraying [4]. However, the filter papers are clearly visible to sprayers and this could cause behavioural bias, and also no post-spray measurements are possible for estimating decay rates. In the current study, small felt pads were applied pre-spray. It is unlikely that these pads cause the sprayers to bias their behaviour, as they were small and discrete. HPLC validation notwithstanding, the overall, a high quality of spraying was evident with only 4 houses (Houses 1, 5, 12 and 17) clearly undersprayed i.e. below the 20 mg AI/m^2^ target dose. Triplicate samples at high, medium and low levels were taken to evaluate high differences in spraying – for example low levels might be expected to have higher doses due to insecticide run-off. There was some clear level of heterogeneity in lambda cyhalothrin content, although it was not associated with spray height. Interestingly a high variation in spray dosage was recently reported in DDT trials in Gambia [21]. What effect this patchy spraying has on IRS efficacy is not known. There is a strong need for further research which correlates spray quality with insecticidal efficacy in the field, which could be facilitated with the use of the IQKs tested here.

Regardless of the materials used to sample insecticide, methods applied pre-spray are potentially visible to sprayers. Therefore, post-spray removal, which allows truly anonymous sampling, was examined (using the felt pads applied pre-spray as a baseline). The adhesive of Sellotape proved to lift very low quantities of insecticide. This is consistent with the generally low extraction efficiency of Sellotape (~10% of applied insecticide) [7]. While the use of Sellotape has proven tractable for extracting poorly absorbent wettable powder mixtures such as DDT formulations [6], it is likely that the capsule suspension of the ICON 10CS affected the utility of this method. The felt pads, on the other hand, were designed for the automobile industry and contained a strong adhesive backing which was able to effectively sample the insecticide. As a rough estimate the pad adhesive pulls 60 – 80% of insecticide off surfaces, depending on surface type. It should be noted, however, that the bamboo surfaces common on Tanna Island were especially conducive to adhesive extraction. Mud surfaces, more common on the African continent, are likely to be less tractable.

The results of the IQK were available almost immediately and were easily interpreted by the programme staff. Such immediate knowledge of insecticide quantities enables vector control programmes to address three key operational questions: (1) to verify that sprayers have actually sprayed a house when they have declared having done, and evaluate the spray coverage in each house, (2) to verify effective insecticide coverage rates per area – i.e. whether at least 85% of the houses have been sprayed adequately; and (3) to calculate and compare the insecticide application rates for each area. Further, the availability of such timely information will assist programme managers to report on the quality of their operations. Taking this a step further, it may be possible to use the IQK to monitor the degradation rates of insecticides following IRS. This is not facile since degradation will be affected by numerous factors such as wall surface and external environment factors such as temperature and UV light exposure. However, using the IQK it should be feasible to investigate these and other aspects of spray quality.

## Conclusion

The IQK colorimetric assay proved to be a useful tool that was simple to use unider realistic field conditions. The IQK provided visual evidence of IRS spray dynamics and rapid assessment of spray team performance. Importantly, such information enables a measured response in case of spray failures, allowing decisions to be made to respray, retrain staff or check equipment and insecticide formulations. The assay was more practical than the traditional alternatives—HPLC and entomological bioassays—for quality control of IRS. After more product development, it is suggested that programme managers incorporate the IQK into the annual IRS training and operational proceedures. The IQK colorimetric assay will have direct applications for routine quality control in malaria control programmes globally and has the potential to improve the efficacy of vector control operations.

## Competing interests

The authors declare that they have no competing interests.

## Authors’ contributions

MJIP, TLR and JH designed the study. TLR, JCM, JA, HI and MJIP conducted the field work. JA, JCM, HIs, HK, TE and FO conducted the laboratory work. TLR, JCM and MJIP analyzed the data. TLR, JCM and MJIP wrote the manuscript. All authors read and approved the final manuscript.
